# Unveiling Software Limitations in the Assessment of the Minimum Sectional Area and Volume in Cleft LIP and Palate Patients

**DOI:** 10.3390/life15020226

**Published:** 2025-02-04

**Authors:** Beethoven Estevao Costa, Renato Yassutaka Faria Yaedú, Maísa Pereira-Silva, André Luis da Silva Fabris, Michele Garcia-Usó, Osvaldo Magro Filho, Simone Soares

**Affiliations:** 1 Department of Diagnosis and Surgery, School of Dentistry, São Paulo State University (UNESP), Araçatuba 16010-380, SP, Brazil; beethoven.e.costa@unesp.br; 2 Department of Oral and Maxillofacial Surgery, Craniofacial Anomalies Rehabilitation Hospital (HRAC), University of São Paulo (USP), Bauru 17012-900, SP, Brazil; renatoyaedu@gmail.com (R.Y.F.Y.); michele.garcia@alumni.usp.br (M.G.-U.); sisoares@usp.br (S.S.); 3 Department of Dentistry, Universidade Brasil (UB), Fernandópolis 15600-000, SP, Brazil; andre.fabris@hotmail.com

**Keywords:** airway management, airway remodeling, computer image processing assisted, three-dimensional imaging

## Abstract

The increasing use of cone beam computed tomography (CBCT) has led to a growing demand for DICOM software that enables the assessment and measurement of craniofacial structures. This study aimed to compare the airway volume and the minimum axial area in patients with cleft lip and palate using five different imaging software programs: Dolphin3D, InVivo Dental, ITK Snap, InVesalius, and NemoFAB. Initially, 100 CBCT scans were selected by an examiner, and their corresponding DICOM files were collected. The oropharyngeal segments were delineated following the manufacturer’s guidelines, using two different segmentation techniques: interactive and fixed threshold. The results were analyzed using the Friedman test and Wilcoxon post hoc test, with a 5% significance level for all statistical tests. The findings for both the minimum axial area and total volume revealed that the median values across the software groups were higher than expected, and significant differences were observed when comparing the groups (*p* < 0.001). All five software programs showed notable differences in their outputs. Specifically, a statistically significant difference in volume was found across all groups, except between InVivo and ITK-Snap. It is recommended that pre- and post-treatment comparisons be performed using the same software for consistency.

## 1. Introduction

Individuals with cleft lip and palate (CLP) exhibit a heightened prevalence of skeletal dentofacial deformities, characterized by a class III profile [1,2,3]. Typically, the recommended course of treatment involves orthognathic surgery, incorporating maxillary advancement with or without mandibular retrusion [1,4]. This dentofacial anomaly arises as a result of primary surgeries conducted during childhood, thereby influencing the growth patterns in both transversal and sagittal dimensions of the jaws [5,6].

The deformity induces adverse alterations in nasal morphophysiology, including turbinate hypertrophy, deviated septum, and modifications to the nasal floor [6,7]. These skeletal transformations adversely impact the anatomy and physiology of the upper airways in approximately 60% of individuals with cleft lip and palate (CLP) [7,8]. Consequently, these changes detrimentally affect both breathing and speech by diminishing the configuration of the nasal cavity, thereby elevating airway resistance and contributing to impairments in craniofacial development [9]. In response to these challenges, surgeons have increasingly utilized advanced software to assess the upper airway through cone beam computed tomography (CBCT) in CLP patients. This approach allows for the comprehensive monitoring of patients and facilitates the evaluation of surgical outcomes, ultimately enhancing the management of these complex cases.

The emergence of new technologies has showcased the efficiency of cone beam computed tomography (CBCT) in assessing upper airway pathways, a fact substantiated by its accuracy and reliability, as affirmed by numerous studies [10,11,12,13]. Furthermore, the reconstruction and 3D evaluation of the upper airway have proven crucial for comprehending issues related to breathing and quality of life, establishing a link to craniofacial anatomy [14]. The literature indeed emphasizes the advantages of CBCT over other imaging examinations, including a lower radiation dose, high-quality imaging with multiple slices [15], and enhanced accuracy in delineating soft tissues and empty spaces [16].

CBCT is employed for craniofacial analysis, with valuable data extracted, including the assessment of upper airway volume and the identification of the minimum axial cross-sectional area in axial view [17]. Due to alterations in airway morphology associated with facial and skeletal deformities, there has been a heightened significance placed on diagnosing sleep-related diseases in modern medicine. This emphasis has resulted in the integration of such diagnoses into orthodontic and surgical planning. This incorporation is facilitated by a more thorough analysis of the minimum axial area and its intricate connection with obstructive sleep apnea syndrome [18].

Due to the widespread adoption of CBCT, there has been an increased demand for DICOM file software, facilitating the evaluation of the craniofacial area. According to a previous study [19], several programs, including InVivo Dental, Mimics, Ondemand3D, OsiriX, Dolphin3D, NemoCeph, ITK Snap and InVesalius, have emerged to address this demand. However, within the upper airway field, the literature lacks comprehensive comparisons and analyses of the accuracy of these software applications [20,21,22,23].

Aligned with this context, this study aimed to compare five distinct software programs by evaluating the airway volume and minimum axial area of the airway in patients with cleft lip and palate (CLP) undergoing orthognathic surgery. Hence, the primary objective was to evaluate the accuracy and reproducibility of measurements within each software program and across different programs. The secondary objective focused on assessing the user-friendliness of the interface and the time required for reconstructions.

## 2. Materials and Methods

### 2.1. Subjects

In this study, among the CBCT images of patients aged 18–40 years at the time of imaging at the Hospital for Rehabilitation of Craniofacial Anomalies, University of São Paulo, Bauru, Brazil images initially requested for the specific purpose of diagnosing orthognathic surgery and planning its treatment were selected. The inclusion criteria comprised patients with complete unilateral cleft lip and palate, without any syndromes, and possessing CBCT scans of good quality without significant artifacts. Accordingly, CBCT data of 100 patients were randomly selected. This retrospective study was approved by the International Review Board of the University of São Paulo, Bauru, São Paulo, Brazil (20593219.4.0000.5441).

### 2.2. Study Protocol

The CBCTs were acquired using the iCAT scanner (Imaging Sciences International, Hatfield, PA, USA), following specific technical standards: parameters of 120 kVp, 5 mA, field of view (FOV) of 16 cm by 21 cm and voxel size of 0.3 mm^3^, with data exported in DICOM files.

**Figure 1:**
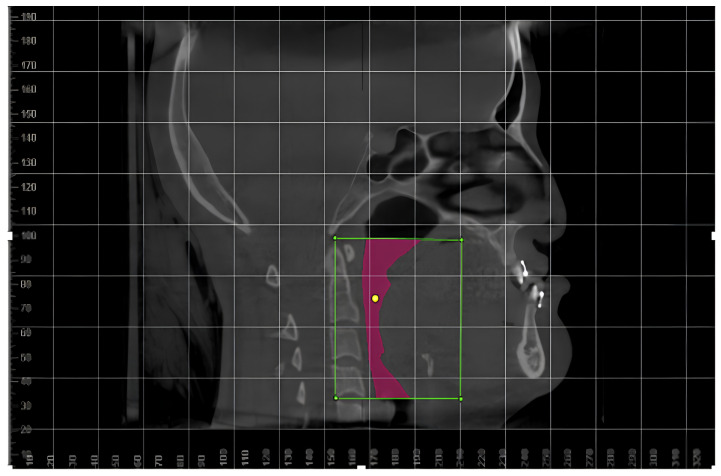
The polygon used for the selection of the region of interest on Dolphin Imaging software (Source: Own authorship, 2022).

An examiner (B.E.C), trained and calibrated, independently analyzed all images in a silent and darkroom setting. For determining the oropharynx volume, three-dimensional volumetric models were constructed and measured using imaging software 17. The anatomical references for oropharynx segmentation included the anteroinferior border of the fourth cervical vertebra (C4), the inferior–posterior boundary of the hyoid bone, the anterior and basal pharyngeal wall, and the upper anterior limit of the palatal bone, forming a rectangle (Figure 1) [21]

Five imaging software programs, each specified in Table 3, were employed to segment and compute volumes from CBCT images. Analyses were conducted using InVivoDental systems (version 5.4, Anatomage, Santa Clara, USA), Dolphin 3D (version 11.7; Dolphin Imaging Systems, CA, USA), ITK (version 3.8.0,18 InVesalius (version 3.1.1, Renato Archer (CTI), Brazil), and NemoCeph 3D-OS (Digital Orthognathic Surgery From Planning to The Surgical Stent, Software Nemotec, Madrid, Spain). Intra-examiner concordance, assessed by repeating CBCT evaluation within a 15-day interval, demonstrated an ICC exceeding 0.8.

Oropharynx segments were delineated following manufacturer’s references, utilizing two segmentation techniques: adjustable threshold range and constant threshold range. Interactive thresholding involved finding the optimal interval based on a visual analysis of oropharynx anatomical limits in axial, sagittal, and coronal sections. The fixed threshold range was set at −1000 to −587 gray levels to assess variability between software programs. Interactive testing segmentation was performed with Dolphin3D, while fixed testing was executed with ITK-Snap, InVivoDental, and InVesalius [20]

**Figure 2:**
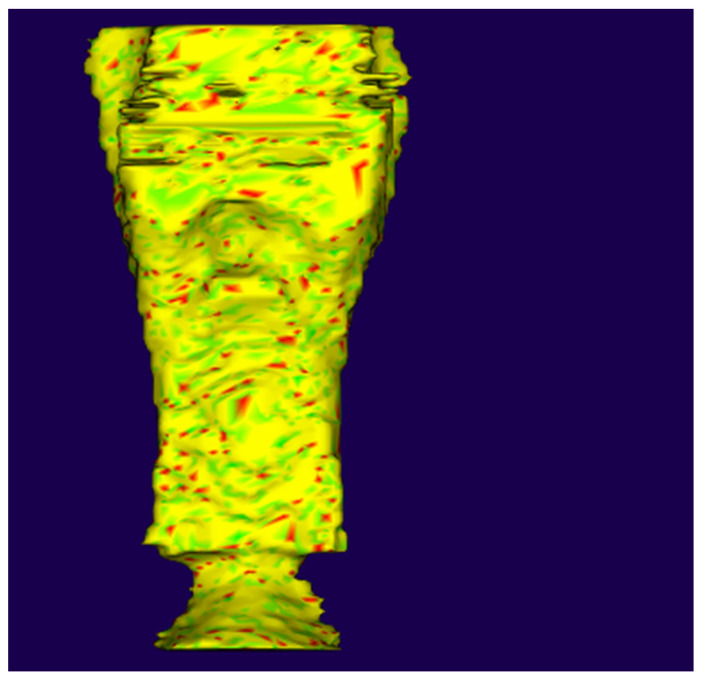
Segmentation from SPHARMPDM module (Source: Own authorship, 2022).

Minimum cross-sectional area calculation was automated by Dolphin3D, InVivoDental, and NemoFab v. 2022 (Nemotec, Madrid, Spain) software. ITK-Snap and InVesalius segmentations were exported to free external software, SlicerCMF with the SPHARMPDM module, for determining mCSA (Figure 2) [21].

### 2.3. Error Analysis

For the calibration process, 30 CBCT images were randomly selected, and the examiner independently conducted measurements of interest using the five programs. The minimum sectional area and upper airway volume were measured, and these measurements were repeated at least 15 days apart. During the second measurement session, all CBCT datasets were analyzed randomly to facilitate a blind assessment, with the observer having no access to previous results. The reliability of the first and second measures was evaluated using the intraclass correlation coefficient (ICC), categorized as poor (ICC < 0.40), fair to good (0.40 ≤ ICC ≤ 0.75), and excellent (ICC > 0.75) following the classification by Walter et al. in 1998 [24].

Only when the intra-examiner agreement exceeded 0.8 were all images analyzed (Table 1).

Additionally, inter-rater reliability between different measurement methods (software) was assessed using ICC. The ICC was calculated for the minimum cross-sectional area (mCSA) and volume measurements across all software analyzed (Dolphin, InVivo, InVesalius, ITK-Snap, NemoCeph).

### 2.4. Statistical Analysis

The data were organized in an Excel spreadsheet (Microsoft, Redmond, Wash), and the normal distribution for the two variables (volume and minimum sectional area) across the five groups (Dolphin, InVivo, ITK-Snap, InVesalius, and NemoCeph) was assessed using the Shapiro–Wilk test. Given the non-parametric nature of the data and the repeated measures design, multiple comparisons were conducted using the Friedman test. A significance level of 5% was employed for statistical significance.

Pairwise comparisons were performed using the Wilcoxon signed-rank test, with Bonferroni correction applied to adjust for multiple comparisons (Table 2).

## 3. Results

In the present study, the intra-examiner error was higher than 0.8 (Table 1).

In the minimum axial area, all software demonstrated statistically significant differences. On the subject of volume, statistically significant differences were observed across all groups, except between the In VIVO and ITK-Snap software. (Table 2)

In this work, five different programs were used to assess the volume of the oropharynx through the segmentation and reconstruction of virtual 3D models. The five programs use semi-automatic and/or automatic segmentation but have different tools and mechanisms for the airway modeling. Amongst the five applications compared in this study, some technical differences were found. (Table 3).

## 4. Discussion

This study aimed to evaluate and compare five different software programs by analyzing the airway volume and the minimum axial area in patients with cleft lip and palate (CLP) undergoing orthognathic surgery. Statistically significant differences were observed in the results of airway volume and the smaller axial area across the programs. Comparing the results obtained in a single program, all the different software showed a good reproducibility and accuracy of measurements. On the other hand, comparisons of results across different software programs were not reliable.

Regarding the minimum axial area, all five software programs showed statistically significant differences. For volume, statistically significant differences were observed across all programs, except between InVivo and ITK-Snap in this analysis. The manipulation of Hounsfield Units (HU) in reconstructions appears to strongly impact the results, indicating the necessity for further studies to elucidate the appropriate HU range when analyzing upper airways.

For a correct assessment of the upper airway, the image segmentation process needs to be performed with great accuracy and a correct selection of the grayscale, thereby providing better image quality [21]. Factors such as tomograph configurations, radiographic management, positioning in the radiographic take, and the entire process of reconstruction and export of the DICOM files directly affect the final quality of the CBCT exam [25,26]. In the present study, the CBCT was performed with the i-CAT scanner, utilizing specific configurations (Imaging Sciences International, Hatfield, PA, USA) under the following technical standards: parameters of 120 kVp, 5 mA, field of view (FOV) 16 cm by 21 cm, and 0.3 mm^3^ voxel, with data exported in DICOM files. It is important to note that certain artifacts can be produced during the tomographic examination, influencing the segmentation process and its precision [21].

Airway segmentation in Dolphin 3D is executed in an objective, simple, and rapid manner, enabling the analysis of the minimum sectional area. The segmentation process involves the addition of initial points that disperse and diffuse within the space predefined by the grayscale. Precise control of this scale is maintained, and once determined, the area is uniformly filled across all planes (axial, sagittal, and coronal). However, it is worth noting that in this segmentation and filling process, there is a potential for exaggeration, leading to exceeding established limits, particularly in morphologically complex areas. A notable drawback with Dolphin is the display of grayscale in its own units, which differ and are incompatible with software using Hounsfield units. An essential improvement for this program involves an update to enhance the entire segmentation process, especially by adapting the grayscale units to be compatible with other tomography analysis programs.

The assessment of upper airways in InVivo Dental is executed with simplicity and efficiency; however, the region of interest is outlined based on the gray scale, which introduces a potential limitation. This approach does not facilitate a comprehensive review of anatomical boundaries, posing challenges in verifying the accuracy of segmentation, as highlighted in a separate study [21]. In contrast, ITK-Snap, available at no cost, exhibits a steeper learning curve compared to Dolphin3D, InVivo Dental, and Nemocph. It is notably adept at constructing 3D models and employs semi-automated segmentation utilizing gray scale. During the filling of the area pre-established by the gray scale, ITK-Snap demonstrates commendable sensitivity, effectively filling even intricate regions [25]

Extensive documentation supports the software’s utility, accuracy, and segmentation capabilities [25]. However, while ITK-Snap generates tomography volume and segmentation information, it relies on other software to obtain the minimum sectional area, which significantly extends the time and complexity involved in acquiring this supplementary information [22]. Furthermore, the three-dimensional model’s surfaces maintain a realistic appearance devoid of automatic smoothing, necessitating refinement if a smoother outcome is desired.

InVesalius and NemoCeph are the two other programs that were used in this study, and they cater to the needs of researchers and clinicians in the field of airway volume assessment, each with its distinct features and usability. InVesalius is a freely available program, albeit challenging to navigate when contrasted with Dolphin3D, InVivo Dental, ITK-Snap, and NemoCeph. It provides the researcher with enhanced control, enabling precise sculpting of the desired airway volume from surrounding 3D structures. Users can adjust brightness and opacity values to effectively remove unwanted voxels before calculating the final airway volume. Additionally, InVesalius enables the modification of limit values to define the range of displayed density values, resulting in a solid volume representation of the airways.

On the other hand, NemoCeph is a paid program known for its user-friendly interface, which simplifies the process for operators. Users can easily mark specific points within the image to define the area of interest, prompting the program to highlight the selected region. Once the airspace is delineated, NemoCeph efficiently calculates both the airway volume and the minimum sectional area [27].

The accuracy of upper airway volume analysis relies on precise segmentation, and its reliability is contingent on the correct acquisition of cone beam computed tomography (CBCT) images, particularly the selection of the gray scale. Achieving a high-quality image depends on the tomograph’s accurate settings, as well as the radiologist’s expertise in patient positioning, volume reconstruction, voxel size, and DICOM file exportation [18]. Several studies highlight that image quality can be affected by factors such as average voxel density, noise, and artifacts that impact tissue density [28,29,30,31].

Notably, CBCT does not adhere to the standard use of Hounsfield units (HU). Its values are influenced by the type of device, the method of image acquisition, and the patient’s positioning [32]. A previous study underscored the need for a meticulous assessment of CBCT, revealing higher Hounsfield coefficient values in CBCT compared to multi-slice CT scans [33]. These factors imply potential influences on the divergent results observed in our research.

In light of the statistical findings in this study, the primary null hypothesis was rejected. A significant limitation emerged in the analysis of both airway volume and the minimum axial area, aligning with this outcome. This limitation underscores the dependence on the accurate segmentation of CBCTs in the presented programs, a concern that resonates with observations in the existing literature [21]. The application of this methodology revealed a noteworthy discrepancy, with variations of up to 16% in the measurement of volume for identical images and up to 53% in the minimum sectional area. This discrepancy is attributed solely to the choice of software utilized.

Additionally, determining the value and position of the minimum cross-sectional area is notably more complex when using open-source software such as ITK-Snap and InVesalius with the SPHARM-PDM module, compared to Dolphin3D and InVivo software. This increased complexity significantly impacts the clinical usability of these programs, particularly given the importance of time as a critical factor for maxillofacial surgeons.

Given the disparities observed among various software programs, as well as the reproducibility within the same software measurements, the authors strongly recommend that comparisons for pre- and post-treatment follow-up be conducted using the same software. This approach helps mitigate potential errors, such as the magnification or minimization of surgical outcomes, ensuring a more accurate assessment.

## 5. Conclusions

When evaluating results within a single software program, all analyzed options demonstrated high reliability and reproducibility, as indicated by the intraclass correlation coefficient (ICC). Conversely, comparisons of results across different software programs lacked reliability. Each software program has its merits and drawbacks. However, considering the parameters utilized in this study, it is incumbent upon the operator, in collaboration with the clinical team, to assess the most suitable software program for the surgical evaluations. Emphasis is placed on the necessity of employing a singular program for both pre- and post-operative assessments.

Each software program has its strengths and limitations, and the choice of the most appropriate tool should be made collaboratively between the operator and the clinical team, considering the specific requirements of surgical evaluations. Importantly, to maintain consistency in pre- and post-operative assessments, using the same software for all measurements is recommended.

A key strength of this study is its inclusion of five different software programs, including cost-effective options, thereby expanding accessibility for clinical and research applications. This demonstrates that a careful selection of tools, combined with proper training and clear communication among the surgical team, can ensure reliable use of these software programs in clinical practice.

## Figures and Tables

**Table 1:** Intra-examiner.

ICC (Intra)		
Software	Minimum Axial Area (mm^2^)	Vol (cm^3^)
Dolphin	0.96	0.96
InVivo	1.00	0.98
InVesalius	0.92	0.92
ITK-Snap	0.81	0.98
NemoCeph	1.00	0.96

ICC, intraclass correlation coefficient.

**Table 2:** Pairwise comparisons using Wilcoxon signed-rank test.

	Minimum Axial Area	Volume
Comparison	*p*-Value	*p*-Value
Dolphin vs. InVivo	6.93 × 10^−17^	7.25 × 10^−16^
Dolphin vs. InVesalius	1.20 × 10^−15^	2.74 × 10^−11^
Dolphin vs. ITK-Snap	2.26 × 10^−12^	6.63 × 10^−15^
Dolphin vs. NemoCeph	2.01 × 10^−14^	6.90 × 10^−4^
InVivo vs. InVesalius	4.96 × 10^−18^	6.20 × 10^−3^
InVivo vs. ITK-Snap	1.37 × 10^−17^	0.462
InVivo vs. NemoCeph	5.87 × 10^−14^	7.55 × 10^−9^
InVesalius vs. ITK-Snap	5.14 × 10^−8^	1.08 × 10^−4^
InVesalius vs. NemoCeph	2.21 × 10^−17^	1.21 × 10^−4^
ITK-Snap vs. NemoCeph	4.10 × 10^−17^	5.98 × 10^−12^

**Table 3:** Technical differences found when using these five applications.

Software	Dolphin3D	InVivoDental	InVesalius	ITK-Snap	NemoFAB
Files	DICOM	DICOM	DICOM	MULTIPLE	DICOM
Segmentation	Quick upper airway segmentation. Good segmentation sensitivity. Segmentation can be checked in 2D slices (axial, coronal, and sagittal).	Quick upper airway segmentation. Threshold interval units (gray levels) compatible with other imaging software.	Segmentation can be checked in 2D slices (axial, coronal, and sagittal).	Segmentation can be checked in 2D slices (axial, coronal, and sagittal). Threshold interval units (gray levels) compatible with other imaging software.	Threshold interval units (gray levels) compatible with other imaging software.
Measurements	Automatically calculates volume, area, and mCSA of the segmentation.	Automatically calculates volume, area, and mCSA of the segmentation.	Automatically calculates volume. To determine area and mCSA, it has to export the file to SlicerCMF in order to create a 3D surface model using the SPHARM-PDM module.	Automatically calculates volume. To determine area and mCSA, it has to export the file to SlicerCMF in order to create a 3D surface model using the SPHARM-PDM module.	Automatically calculates volume, area, and mCSA of the segmentation.
3D visualization	Shows an automatic 3D rendering from the CBCT.	Shows an automatic 3D rendering from the CBCT.	Shows volumetric models only from the segmented structures.	Shows volumetric models only from the segmented structures.	Shows an automatic 3D rendering from the CBCT.
Saving methods	Saves the volumetric models in the software. Not possible to export data.	Saves the volumetric models in the software. Not possible to export data.	Volumetric models can be saved as independent files and exported to different software.	Volumetric models can be saved as independent files and exported to different software.	Saves the volumetric models in the software. Not possible to export data.

## Data Availability

The original contributions presented in the study are included in the article, and further inquiries can be directed to the corresponding authors.
